# Characteristics of Hamstring Electromyographic Activity and the Break-Point Angle during Nordic Hamstring Exercise at Different Ankle Positions

**DOI:** 10.5114/jhk/208232

**Published:** 2025-09-23

**Authors:** Taspol Keerasomboon, Parunchaya Jamkrajang, Weerawat Limroongreungrat, Thammanunt Chrunarm, Toshiaki Soga, Norikazu Hirose

**Affiliations:** 1 College of Sports Science and Technology, Mahidol University, Bangkok, Thailand.; 2 Graduate School of Engineering and Science, Shibaura Institute of Technology, Tokyo, Japan.; 3 Research Fellow of Japan Society for the Promotion of Science, Japan.; 4 Faculty of Sport Sciences, Waseda University, Tokyo, Japan.

**Keywords:** injury prevention, hamstring muscles, electromyography

## Abstract

Nordic hamstring exercise (NHex) is well-known to reduce hamstring injury risk. However, the semitendinosus (ST) muscle is more activated than the biceps femoris long head (BFlh) muscle during NHex, though the BFlh muscle is more vulnerable to injury. It is important to investigate different NHex settings that may increase its effectiveness. This study aimed to examine the electromyographic (EMG) activity of the hamstring muscles and the break-point angle (BPA) during the NHex with the ankle joint positioned in plantar flexion (PF), dorsiflexion (DF) (neutral), and DF (neutral) with support. Twelve male volunteers without hamstring injuries in the four years preceding the experiment (age: 20.58 ± 0.9 years; body height: 171.1 ± 7.8 cm; body mass: 66.9 ± 12.2 kg) took part in the experiment. Participants randomly performed two sessions of the following exercise tests: NHex with ankle DF, NHex with ankle DF with sole support (DFS), and NHex with ankle PF. The EMG activity of the hamstring, BFlh, and ST muscles was measured for both the dominant and non-dominant limbs. The BPA was recorded using an IMU sensor. A repeated measures analysis of variance was conducted to assess hamstring muscle activity and the BPA. There was a significant main effect of EMG activity (p < 0.05) during the NHex under different ankle joint conditions for the BFlh in both the dominant and non-dominant legs. The findings indicated that EMG activity significantly increased during PF (p < 0.05) relative to DF and DFS for the BFlh in the dominant leg. Additionally, there were no significant differences in the BPA across different ankle positions (p > 0.05). This study demonstrated that the NHex elicited greater BFlh activity in PF than in DF and DFS.

## Introduction

Hamstring strain injury (HSI) is among the most common sports injuries and occurs during high-intensity sprinting (Bourne et al., 2018; Erickson and Sherry, 2017). The re-injury rate of HSI is approximately 30% (Heiser et al., 1984; Orchard and Best, 2002). Consequently, preventing hamstring injury and its recurrence is essential for maintaining performance in athletes.

In high-speed running, HSI usually happens during the late swing phase (Schache et al., 2009). About 80% of all HSIs occur in the biceps femoris long head (BFlh) muscle, which is more susceptible to injury than other biarticular hamstring muscles, including the semitendinosus (ST) and the semimembranosus (SM) (Bourne et al., 2016; Brosseau et al., 1997; Ditroilo et al., 2013). During the initial stance phase or terminal swing, HSI mostly affects the BFlh muscle. During the late swing phase, BFlh electromyographic (EMG) activity exceeds that observed at other stages (Higashihara et al., 2015). Additionally, during the initial stance phase, BFlh EMG activity is greater than ST EMG activity (Higashihara et al., 2018). These characteristics suggest a potential mechanism for HSI.

A key strategy for preventing HSI is to enhance strength during eccentric contraction of the hamstring muscles (Franca et al., 2024; Opar et al., 2015; Timmins et al., 2016; van der Horst et al., 2015). The Nordic hamstring exercise (NHex) is commonly prescribed to reduce hamstring injury risk (Bourne et al., 2017a, 2018; van Dyk et al., 2019; Zabaloy et al., 2025). This NHex, highlighting eccentric contraction, has demonstrated a reduction in HSI incidence among athletes (van Dyk et al., 2019), increases in eccentric strength, and beneficial architectural alterations in the hamstrings by enhancing fascicle length (Bourne et al., 2017a, 2017b, 2018; Cuthbert et al., 2020). Nonetheless, numerous studies have highlighted limitations of the NHex (Ditroilo et al., 2013; Hirose et al., 2021; Šarabon et al., 2019; Soga et al., 2021). The break-point angle (BPA) is defined as the position in which the athlete can no longer resist this external knee flexion load, resulting from forward trunk inclination (Soga et al., 2021, 2022). The BPA of the conventional NHex has been reported as of approximately 60° (Soga et al., 2021), possibly because of greater ST activation compared to BFlh activation, despite the BFlh’s increased susceptibility to injury (Bourne et al., 2017b, 2018; Fernandez-Gonzalo et al., 2016; Hirose et al., 2021; Soga et al., 2021).

Other factors influencing EMG activity patterns include muscle length, morphology, and the number of recruited joints (Hirose and Tsuruike, 2018; Hirose et al., 2021; Keerasomboon et al., 2020; Mohamed et al., 2002). Previous studies have examined the effects of muscle length changes by adjusting knee flexion and hip angles (Hirose et al., 2021; Keerasomboon et al., 2020, 2022; Šarabon et al., 2019). Performing the NHex at a shallow knee flexion angle increased BFlh activation due to muscle elongation (Hirose et al., 2021). Furthermore, a prior study indicated that ST peak EMG activation was significantly higher than that of the BFlh during the NHex. However, the difference was smaller—or BFlh activity exceeded ST activity—when the hamstrings were elongated using NHex with a flexed hip (Marušič and Šarabon, 2020; Šarabon et al., 2019). However, influence of the ankle position on hamstring EMG activity remains unclear.

The ankle position has been identified as a critical technical variable influencing the NHex (Comfort et al., 2017). Furthermore, a prior study demonstrated that modifying the ankle position affected the gastrocnemius and the force-length relationship. This modification enhances the gastrocnemius’ force-generating capacity and contributes to knee flexor torque (Vicente-Mampel et al., 2022). Only few studies have evaluated the impact of ankle positioning, such as dorsiflexion (DF) and plantar flexion (PF), during the NHex, with findings indicating no substantial differences in BFlh and ST activity with alterations in the position of the ankle (Comfort et al., 2017; Vicente-Mampel et al., 2022). A limitation of the previous research was that hamstring muscle sEMG activity was normalized using only a 45° knee flexion angle (Vicente-Mampel et al., 2022). Normalization was dependent on the angle of the knee joint (Onishi et al., 2002). Leg curls performed with the angle of knee flexion ranging from 30° to 60° predominantly stimulate the BFlh, whereas the ST exhibits greater activation than the BFlh at knee flexion angles exceeding 60° (Hirose and Tsuruike, 2018). Furthermore, only one study has reported no effect of the ankle position on gastrocnemius activation (Comfort et al., 2017). Ultimately, various methods exist for identifying BPAs (Sconce et al., 2021). The BPA has been defined as the point at which individuals could no longer maintain the required descent tempo (10°/s), and hamstring EMG activity may decline after reaching the BPA during the NHex (Soga et al., 2021). Therefore, the effects of the ankle position on hamstring EMG activity and the BPA remain unclear. This study aimed to investigate the EMG activation of the hamstring muscles and the BPA at different ankle positions during the NHex. It was hypothesized that PF during the NHex would result in greater BFlh activation.

## Methods

### Participants

The sample size was calculated using G*Power 3.1.3 software (Heinrich Heine Universität Düsseldorf, Germany). A two-way repeated measures analysis of variance was conducted, with a significance level of 0.05 and statistical power set to 0.8. Based on this calculation, a minimum sample size of 12 participants was deemed sufficient. Accordingly, 12 male non-competitive athletes (age: 20.58 ± 0.9 years; body height: 171.1 ± 7.8 cm; body mass: 66.9 ± 12.2 kg, all reported as mean ± standard deviation) participated in this study.

None of the participants had a history of HSI within the four years prior to the experiment. Participants were excluded if they were unable to perform the NHex because of a current injury to the lower and/or upper extremities. Additionally, participants with ACL injuries were excluded. The NHex was not part of the participants' regular resistance training program. The study protocol received approval from the institutional review board of the Mahidol University, Nakhon Pathom, Thailand (approval number: MU-CIRB 2023/226.1407; approval date: 21 July 2023), and all procedures were conducted in compliance with the Declaration of Helsinki. All participants were informed of the study's objectives and methodology, and formal agreement was acquired.

### Design and Procedures

A crossover design was used in this study to examine EMG activity and the BPA of the hamstring muscles during the NHex with various ankle positions.

Prior to the experiment, participants completed warm-up activities and were prepared for surface EMG electrode placement. Hair surrounding the target areas was shaved, and the skin was cleaned with alcohol to minimize noise. The electrodes were affixed to seven target muscles: BFlh, ST, gluteus maximus (GM), rectus abdominis (RA), erector spinae (ES), lateral gastrocnemius (LG), and medial gastrocnemius (MG).

Following electrode placement, participants executed maximal voluntary isometric contractions (MVICs) to normalize subsequent muscle EMG recordings. Participants executed two repetitions of the prone leg curl at 30° and 90° of knee flexion with MVIC, utilizing manual resistance for the BFlh and ST muscles. This study utilized prone leg curls at 30° and 90° because previous research suggested that knee flexion angles between 30° and 60° would preferentially stimulate the BFlh, whereas ST activation would be greater at angles exceeding 60° (Hirose and Tsuruike, 2018). To assess the MG and LG muscles, participants stood on one leg and performed PF of the foot. The GM was evaluated with the participant lying prone, lifting the entire leg against manual resistance. For the ES muscle, participants elevated their trunk from a prone position while resisting manual force. Finally, participants performed abdominal curls against manual resistance to assess RA activity. These MVIC protocols have been validated in earlier studies to normalize EMG activity for the specified muscles (Hirose and Tsuruike, 2018; Keerasomboon et al., 2020). Each MVIC protocol was conducted for 5 s per repetition, and EMG data were recorded throughout. After the examiner verified high-quality EMG signals for all target muscles during MVIC trials, participants performed a single submaximal NHex trial as a familiarization effort.

**Figure 1:**
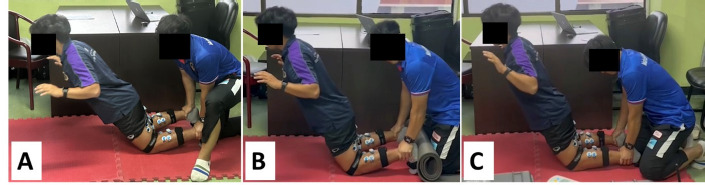
. Variations of the (NHex). (A) NHex with ankle dorsiflexion (DF), (B) NHex with ankle dorsiflexion with sole support (DFS), and (C) NHex with ankle plantarflexion (PF).

For the NHex protocol, participants started the exercise in a kneeling position, with their hands extended in front of them and their elbows fully flexed. The examiner securely held the participant’s ankle against the horizontal or sloped platform while instructing the participant to maintain a straight alignment from the knees to the head. The examiner then directed participants to lean forward as slowly as possible. Following the familiarization protocol, two repetitions of the NHex with variations in the ankle position were performed in randomized order: (1) NHex with ankle DF, (2) NHex with ankle DF with sole support (DFS), and (3) NHex with ankle PF (Figure 1). Participants were given a minimum of 3 min to rest between repetitions and conditions. The EMG data acquired under each condition were evaluated and standardized to the values recorded during the MVIC of each muscle (normalized electromyography [nEMG]). The experiments were performed under the guidance of a certified examiner from the Australian Strength and Conditioning Association.

**Figure 2:**
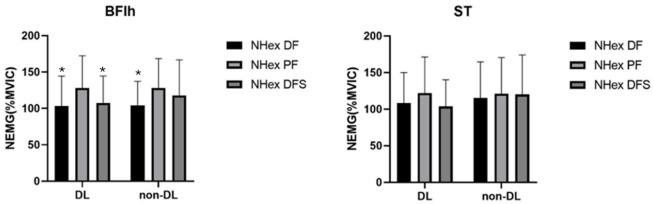
Electromyographic (EMG) activity of the biceps femoris long head (BFlh) and the semitendinosus (ST) in both the dominant (DL) and the non-dominant leg (non-DL) during Nordic hamstring exercise (NHex) variations: NHex with ankle dorsiflexion (NHex DF), NHex with ankle dorsiflexion with sole support (NHex DFS), and NHex with ankle plantarflexion (NHex PF). * a significant difference was observed for NHex with ankle plantarflexion (p < 0.05)

**Figure 3:**
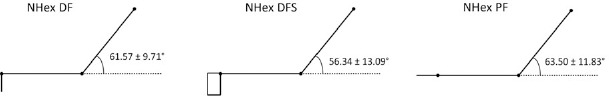
Break-point angle during variations of the Nordic hamstring exercise (NHex): NHex with ankle dorsiflexion (NHex DF), NHex with ankle dorsiflexion with sole support (NHex DFS), and NHex with ankle plantarflexion (NHex PF).

**Figure 4:**
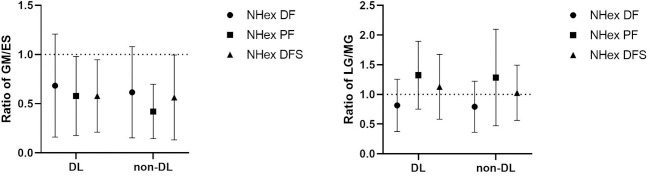
Ratio of the gluteus maximus (GM) to the erector spinae (ES) and the lateral gastrocnemius (LG) to the medial gastrocnemius (MG) in both the dominant (DL) and the non-dominant leg (non-DL) during Nordic hamstring exercise (NHex) variations: NHex with ankle dorsiflexion (NHex DF), NHex with ankle dorsiflexion with sole support (NHex DFS), and NHex with ankle plantarflexion (NHex PF). In the left graph, a GM/ES ratio below 1.0 indicates that the normalized electromyographic (nEMG) activity of the erector spinae (ES) was higher than that of the gluteus maximus (GM) (dashed horizontal line). In the right graph, an LG/MG ratio above 1.0 indicates that the nEMG activity of the lateral gastrocnemius (LG) was higher than that of the medial gastrocnemius (MG) (dashed horizontal line).

**Figure 5:**
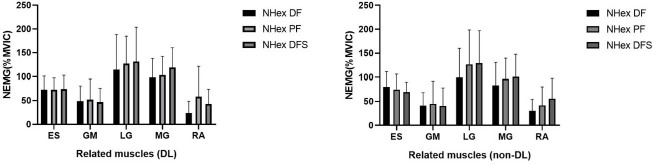
Electromyographic (EMG) activity of the erector spinae (ES) in both the dominant (DL) and the non-dominant leg (non-DL) during Nordic hamstring exercise (NHex) variations: NHex with ankle dorsiflexion (NHex DF), NHex with ankle dorsiflexion with sole support (NHex DFS), and NHex with ankle plantarflexion (NHex PF).

### Electromyography

The sampling rate of the EMG signal was at 2,000 Hz using a wireless surface EMG system (Noraxon, Scottsdale, AZ, USA). Measurements were conducted utilizing circular bipolar surface electrodes on both legs (Ambu®, type Blue Sensor P-00-S/50, Ag/AgCl, diameter: 13 mm, center-to-center distance: 25 mm, Ballerup, Denmark). The EMG electrodes were pre-amplified (10×) and connected through the EMG mainframe, and the signal was band-pass filtered (20–450 Hz). To prepare for data collection, the skin was cleaned with alcohol wipes and shaved as needed to enhance sensor adherence and minimize noise. The electrodes were positioned on each designated muscle according to the following anatomical landmarks: for the BFlh, the electrode was placed in the middle of the ischial tuberosity and the lateral epicondyle of the tibia; for the ST, at the midpoint of the line connecting the ischial tuberosity and the medial epicondyle of the tibia; for the SM, the electrode was located along the line between the medial condyle of the tibia and the ischial tuberosity; for the GM, at the point that was halfway between the sacral vertebrae and the greater trochanter; for the ES, the electrode was placed two finger widths lateral to the spinous process of the L1 vertebra; for the RA, two finger widths to the side of the umbilicus, which was the midway of the line; for the MG, the electrode was placed at the midpoint of the most noticeable bulge of the muscle between the proximal and the posterior part of the medial condyle adjacent to the femur, the knee joint capsule, and the middle part of the posterior surface of the calcaneus; for the LG, the electrode was located one-third along the line between the head of the fibula and the heel. All electrodes were positioned parallel to the lines connecting these landmarks, as suggested by the SENIAM guidelines (Hermens et al., 2000). To ensure accurate electrode positioning, the examiner conducted palpation of the muscle bellies and an EMG assessment.

### Kinematic Data

Kinematic data were recorded during the tasks using inertial measurement unit (IMU) sensors (MyoMOTION Research Sensors, Noraxon U.S.A., Scottsdale, Arizona, USA). Participants were equipped with IMU sensors on the pelvis, left and right thighs, left and right shanks, and left and right feet. The sampling rate was set at 100 Hz. The IMU sensors were affixed to the pelvis at the sacral location, to the thigh, at the anterior and distal segments, secured with elastic straps, to the shank, they were anterior and slightly medial along the tibia, and to the upper foot, beneath the ankle, secured with a bandage.

### Data Analysis

For EMG data, MR 3.14 MyoResearch software (Noraxon, Scottsdale, AZ, USA) was used to analyze EMG activity. The raw EMG data were band-pass filtered (20–450 Hz) and processed using the root mean square (RMS) over a 100-ms window. The RMS value during 2 s of the 5-s MVIC trials was calculated as the mean (Hirose et al., 2021). The mean EMG activity of two valid repetitions from each task was used for further analysis. The RMS of the EMG data from each condition was normalized to the values recorded during the MVIC of each muscle (nEMG). The %MVIC for each NHex variation was calculated by dividing the RMS of each NHex variation by the mean MVIC value.

The IMU-based body model for joint angle calculations was established using MyoResearch 3 (MR3; Noraxon, Scottsdale, AZ, USA). Kinematic data were obtained from the relationships between various right-handed Cartesian coordinate systems: x-axis: directed along the length of the IMU towards the top; y-axis: oriented to the left of the IMU; and z-axis: extending outward, perpendicular to the IMU surface. The joint angle decomposition sequences in MR3 adhered to the International Society of Biomechanics guidelines (Wu et al., 2002). The lower extremity joint angles recorded in MR3 were exported and imported into Visual3D software for further BPA analysis. The angular velocity of knee extension was determined by dividing the angle of knee flexion by the time interval. The BPA was characterized as the angle at which the angular velocity of knee extension went over 10°/s (Soga et al., 2022).

### Statistical Analysis

Values are expressed as mean ± standard deviation. The Shapiro-Wilk test was used and confirmed normal distribution. A repeated-measures analysis of variance was conducted to examine the NHex changes (DF, PF, and DFS) as a within-participant factor and the other muscles as a between-participant factor. A one-way repeated-measures analysis of variance (DF, PF, and DFS) was used to compare BPA levels during NHex variations. Statistical significance was determined at a level of *p* < 0.05. Based on the significance of the main or interaction effects, a Bonferroni post hoc test was performed. The partial η^2^ was categorized according to the following effect size criteria: trivial < 0.01, moderate 0.01–0.06, medium 0.06–0.14, and large > 0.14. Cohen’s *d* was categorized according to the following effect size criteria: trivial < 0.2, small 0.2–0.5, medium 0.5–0.8, and large > 0.8. The significance threshold was set at *p* < 0.05. Statistical analyses were conducted using JASP (version 0.19.1).

## Results

### Hamstring EMG Activity

There was a significant main effect in nEMG activity during the NHex with different ankle joint conditions for the BFlh in the dominant leg (DL) (F [2,22] = 9.26, partial η^2^ = 0.45, *p* = 0.001) and the non-DL (F [2,22] = 4.4, partial η^2^ = 0.28, *p* = 0.025). The results revealed that nEMG activity under the PF condition was significantly greater than under the DF (mean difference: 24.77%, 95% CI [4.4%, 45.1%]; *d* = 0.6; *p* = 0.017) and DFS (mean difference: 20.42%, 95% CI [7.6%, 33.2%]; *d* = 0.49; *p* = 0.003) conditions in the DL. For non-DL participants, PF was significantly higher than DF alone (mean difference: 23.86%, 95% CI [0.4%, 47.2%]; *d* = 0.57; *p* = 0.045). No significant difference was observed in the ST (*p* > 0.05) under any condition. Moreover, no significant difference was observed in the ST/BF ratio (*p* > 0.05) under any condition.

### BPA

The BPA levels did not exhibit significant differences (*p* > 0.05) under any of the studied conditions.

### Related Muscles

No significant differences were observed in the ratios of GM/ES, LG/MG, or related muscles (*p* > 0.05) under any of the conditions considered in this study.

## Discussion

This study examined the hamstring and related muscles activity during the NHex with the adjustment of ankle joint positions at PF, DF (neutral), and DF (neutral) with support in both the DL and non-DL groups. Our findings demonstrated the influence of various ankle positions (i.e., PF vs. DF) on BFlh activation, whereas ST activation remained unaffected. Furthermore, the BPA was not influenced by changes in the ankle position during the NHex.

The primary finding was that nEMG of the BFlh was predominantly recruited during NHex PF, rather than DF and DFS. This finding contradicts previous research, which demonstrated no significant differences in biceps femoris activation with alterations in the ankle position during the NHex (Vicente-Mampel et al., 2022). This study, however, did not identify a clear underlying mechanism for this result. As reported by Vicente-Mampel et al. (2022), one possible mechanism could be that they normalized BFlh and ST sEMG activity using only a 45° knee joint angle, whereas in our study we used 30° to normalize the BFlh and 90° to normalize the ST, based on another prior study (Hirose and Tsuruike, 2018). Leg curls performed at knee flexion angles between 30° and 60° predominantly activate the BFlh, whereas ST activation surpasses BFlh activation at knee flexion angles exceeding 60° (Hirose and Tsuruike, 2018). Moreover, normalization is dependent on the knee joint angle (Onishi et al., 2002). The discrepancies may be attributed to changes in MVIC protocols. Another possible explanation is that altering the ankle position affects gastrocnemius muscle length. Anatomically, the gastrocnemius muscle originates from the medial and lateral epicondyles of the femur (Comfort et al., 2017). Because of its fascial connections with the hamstring muscles, the gastrocnemius may influence force production via its inherent length-tension characteristics (Comfort et al., 2017). It is possible that PF of the ankle minimizes gastrocnemius involvement in knee joint torque regulation while significantly increasing the contribution of the biceps femoris, which has a larger physiological cross-sectional area than that of the ST and is particularly important for force generation.

This study found no differences in the BPA among the different ankle variations. It has been shown that the BPA of the conventional NHex is approximately 60° (Soga et al., 2021), which is in line with our results (61.5° for the NHex with DF). The BPA indicates that the hamstrings do not experience an adequate eccentric load stimulus during the final period of descent. It has been proposed that hamstring EMG activity decreases following the BPA (Monajati et al., 2017). Moreover, hamstring EMG activity is reduced after the BPA during the standard NHex (Soga et al., 2021). The BPA during the NHex corresponds to the quasi-isometric hamstring movement observed in the late swing phase (Van Hooren and Bosch, 2017). This quasi-isometric mechanism may mitigate muscle damage and strain injury (Van Hooren and Bosch, 2017). However, our findings indicate that varying ankle positions (i.e., PF vs. DF) do not affect the BPA, which is inconsistent with a previous study by Vicente-Mampel et al. (2022). DF resulted in a significantly greater BPA than that of PF. One possibility is that our study determined the BPA as the angle at which the angular velocity of knee extension exceeded 10°/s, determined by earlier studies (Soga et al., 2021, 2022). The BPA has been defined as the point of greatest angular acceleration of the knee, where individuals can no longer withstand the rising gravitational moment and descend to the ground (Vicente-Mampel et al., 2022). Different methods for determining the BPA may lead to variations in results (Sconce et al., 2021). Moreover, in the Vicente-Mampel et al.’s (2022) study, BPA values for NHex PF and NHex DF were approximately 29.7° and 36.4°, respectively, and were significantly lower than those of our participants (63.5° and 61.5° for PF and DF, respectively). These differences may be attributed to participants’ backgrounds as our study sample consisted of healthy, untrained individuals, whereas in the Vicente-Mampel et al.’s (2022) study, participants were field hockey players.

Our results showed absolutely no impact of varied ankle positions (i.e., PF vs. DF) on the GM/ES, LG/MG ratios, or related muscles. However, we could not elucidate the underlying mechanisms associated with this result. One possible explanation is that BPA values for all conditions were similar, at approximately 60°. As participants leaned forward in the NHex, the ES muscles resisted hip extension torque. Furthermore, to reduce the distance between the ES and the ground, the ES was activated to maintain an upright trunk posture opposing gravitational force and hip extension torque exceeding the GM (Narouei et al., 2018). Additionally, our findings align with those of another study (Narouei et al., 2018), which reported that during the NHex, the demand for ES activity exceeded that of the GM. For the MG, as speculated in prior research (Comfort et al., 2017) which aligns with our findings, no significant variations in MG activation were observed in either variation of the NHex when performed with the ankle in DF or PF. We found only one previous study that examined MG activity; however, the underlying mechanisms remain unclear.

This study has several limitations. First, the ankle angle was not measured, limiting our ability to clarify the underlying mechanism. Second, all participants were men, making it unclear what hamstring EMG activity would be in female participants. Finally, actual muscle forces were not assessed. Despite these limitations, the results and conclusions of this study have practical implications. Performing the NHex in PF preferentially recruited the BFlh compared to other conditions, which may have positive effects on HSI prevention. However, future studies are necessary to validate the hypothesis that PF leads to greater BFlh activation.

## Conclusions

This study showed that NHex performed in the PF position is preferable for recruiting BFlh activity compared to the NHex performed in DF with or without sole support. The NHex in the PF position may provide a more favorable setting for BFlh activation. The results of this study may assist coaches and healthcare professionals in designing programs that effectively target BFlh activation, which is crucial for HSI prevention by adjusting the ankle position during the NHex.

## References

[ref1] Bourne, M. N., Duhig, S. J., Timmins, R. G., Williams, M. D., Opar, D. A., Al Najjar, A., Kerr, G. K., & Shield, A. J. (2017a). Impact of the Nordic hamstring and hip extension exercises on hamstring architecture and morphology: implications for injury prevention. *British Journal of Sports Medicine*, 51(5), 469–477. 10.1136/bjsports-2016-096130 27660368

[ref2] Bourne, M. N., Opar, D. A., Williams, M. D., Al Najjar, A., & Shield, A. J. (2016). Muscle activation patterns in the Nordic hamstring exercise: Impact of prior strain injury. *Scandinavian Journal of Medicine & Science in Sports*, 26(6), 666–674. 10.1111/sms.12494 26059634

[ref3] Bourne, M. N., Timmins, R. G., Opar, D. A., Pizzari, T., Ruddy, J. D., Sims, C., Williams, M. D., & Shield, A. J. (2018). An Evidence-Based Framework for Strengthening Exercises to Prevent Hamstring Injury. *Sports Medicine*, 48(2), 251–267. 10.1007/s40279-017-0796-x 29116573

[ref4] Bourne, M. N., Williams, M. D., Opar, D. A., Al Najjar, A., Kerr, G. K., & Shield, A. J. (2017b). Impact of exercise selection on hamstring muscle activation. *British Journal of Sports Medicine*, 51(13), 1021–1028. 10.1136/bjsports-2015-095739 27467123

[ref5] Brooks, J. H., Fuller, C. W., Kemp, S. P., & Reddin, D. B. (2006). Incidence, risk, and prevention of hamstring muscle injuries in professional rugby union. *American Journal of Sports Medicine*, 34(8), 1297–1306. 10.1177/0363546505286022 16493170

[ref6] Brosseau, L., Tousignant, M., Budd, J., Chartier, N., Duciaume, L., Plamondon, S., O'Sullivan, J. P., O'Donoghue, S., & Balmer, S. (1997). Intratester and intertester reliability and criterion validity of the parallelogram and universal goniometers for active knee flexion in healthy subjects. *Physiotherapy Research International: The Journal for Researchers and Clinicians in Physical Therapy*, 2(3), 150–166. 10.1002/pri.97 9421820

[ref7] Comfort, P., Regan, A., Herrington, L., Thomas, C., McMahon, J., & Jones, P. (2017). Lack of Effect of Ankle Position During the Nordic Curl on Muscle Activity of the Biceps Femoris and Medial Gastrocnemius. *Journal of Sport Rehabilitation*, 26(3), 202–207. 10.1123/jsr.2015-0130 27632836

[ref8] Cuthbert, M., Ripley, N., McMahon, J. J., Evans, M., Haff, G. G., & Comfort, P. (2020). The Effect of Nordic Hamstring Exercise Intervention Volume on Eccentric Strength and Muscle Architecture Adaptations: A Systematic Review and Meta-analyses. *Sports Medicine*, 50(1), 83–99. 10.1007/s40279-019-01178-7 31502142 PMC6942028

[ref9] Ditroilo, M., De Vito, G., & Delahunt, E. (2013). Kinematic and electromyographic analysis of the Nordic Hamstring Exercise. *Journal of Electromyography and Kinesiology*, 23(5), 1111–1118. 10.1016/j.jelekin.2013.05.008 23809430

[ref10] Ekstrand, J., Hägglund, M., & Waldén, M. (2011). Injury incidence and injury patterns in professional football: the UEFA injury study. *British Journal of Sports Medicine*, 45(7), 553–558. 10.1136/bjsm.2009.060582 19553225

[ref11] Erickson, L. N., & Sherry, M. A. (2017). Rehabilitation and return to sport after hamstring strain injury. *Journal of Sport and Health Science*, 6(3), 262–270. 10.1016/j.jshs.2017.04.001 30356646 PMC6189266

[ref12] Fernandez-Gonzalo, R., Tesch, P. A., Linnehan, R. M., Kreider, R. B., Di Salvo, V., Suarez-Arrones, L., Alomar, X., Mendez-Villanueva, A., & Rodas, G. (2016). Individual Muscle use in Hamstring Exercises by Soccer Players Assessed using Functional MRI. *International Journal of Sports Medicine*, 37(7), 559–564. 10.1055/s-0042-100290 27116347

[ref13] França, C., Martins, F., Przednowek, K., Marques, A., Ihle, A., Sarmento, H., Gouveia, É. R. (2024). Knee and Hip Muscle Strength of Male Soccer Players from Different Competitive Levels. *Journal of Human Kinetics*, 93, 17–27. 10.5114/jhk/185217 39132414 PMC11307174

[ref14] Heiser, T. M., Weber, J., Sullivan, G., Clare, P., & Jacobs, R. R. (1984). Prophylaxis and management of hamstring muscle injuries in intercollegiate football players. *American Journal of Sports Medicine*, 12(5), 368–370. 10.1177/036354658401200506 6496833

[ref15] Hermens, H. J., Freriks, B., Disselhorst-Klug, C., & Rau, G. (2000). Development of recommendations for SEMG sensors and sensor placement procedures. *Journal of Electromyography and Kinesiology*, 10(5), 361–374. 10.1016/s1050-6411(00)00027-4 11018445

[ref16] Higashihara, A., Nagano, Y., Ono, T., & Fukubayashi, T. (2015). Differences in activation properties of the hamstring muscles during overground sprinting. *Gait & Posture*, 42(3), 360–364. 10.1016/j.gaitpost.2015.07.002 26213185

[ref17] Higashihara, A., Nagano, Y., Ono, T., & Fukubayashi, T. (2018). Differences in hamstring activation characteristics between the acceleration and maximum-speed phases of sprinting. *Journal of Sports Sciences*, 36(12), 1313–1318. 10.1080/02640414.2017.1375548 28873030

[ref18] Hirose, N., & Tsuruike, M. (2018). Differences in the Electromyographic Activity of the Hamstring, Gluteus Maximus, and Erector Spinae Muscles in a Variety of Kinetic Changes. *Journal of Strength and Conditioning Research*, 32(12), 3357–3363. 10.1519/jsc.0000000000002747 30102684

[ref19] Hirose, N., Tsuruike, M., & Higashihara, A. (2021). Biceps Femoris Muscle is Activated by Performing Nordic Hamstring Exercise at a Shallow Knee Flexion Angle. *Journal of Sports Science & Medicine*, 20(2), 275–283. 10.52082/jssm.2021.275 34211320 PMC8219264

[ref20] Keerasomboon, T., Mineta, S., & Hirose, N. (2020). Influence of Altered Knee Angle and Muscular Contraction Type on Electromyographic Activity of Hamstring Muscles during 45° Hip Extension Exercise. *Journal of Sports Science & Medicine*, 19(4), 630–636. 33239935 PMC7675620

[ref21] Keerasomboon, T., Soga, T., & Hirose, N. (2022). Influence of Altered Knee Angle on Electromyographic Activity of Hamstring Muscles Between Nordic Hamstring Exercise and Nordic Hamstring Exercise with Incline Slope Lower Leg Board. *International Journal of Sports Physical Therapy*, 17(5), 832–840. 35949369 10.26603/001c.36627 PMC9340823

[ref22] Marušič, J., & Šarabon, N. (2020). Comparison of electromyographic activity during Nordic hamstring exercise and exercise in lengthened position. *European Journal of Translational Myology*, 30(2), 8957. 10.4081/ejtm.2019.8957 32782761 PMC7385688

[ref23] Mohamed, O., Perry, J., & Hislop, H. (2002). Relationship between wire EMG activity, muscle length, and torque of the hamstrings. *Clinical Biomechanics*, 17(8), 569–579. 10.1016/S0268-0033(02)00070-0 12243716

[ref24] Monajati, A., Larumbe-Zabala, E., Goss-Sampson, M., & Naclerio, F. (2017). Analysis of the Hamstring Muscle Activation During two Injury Prevention Exercises. *Journal of Human Kinetics*, 60, 29–37. 10.1515/hukin-2017-0105 29339983 PMC5765783

[ref25] Narouei, S., Imai, A., Akuzawa, H., Hasebe, K., & Kaneoka, K. (2018). Hip and trunk muscles activity during nordic hamstring exercise. *Journal of Exercise Rehabilitation*, 14(2), 231–238. 10.12965//jer.1835200.600 29740557 PMC5931159

[ref26] Onishi, H., Yagi, R., Oyama, M., Akasaka, K., Ihashi, K., & Handa, Y. (2002). EMG-angle relationship of the hamstring muscles during maximum knee flexion. *Journal of Electromyography and Kinesiology*, 12(5), 399–406. 12223173 10.1016/s1050-6411(02)00033-0

[ref27] Opar, D. A., Williams, M. D., Timmins, R. G., Hickey, J., Duhig, S. J., & Shield, A. J. (2015). Eccentric hamstring strength and hamstring injury risk in Australian footballers. *Medicine and Science in Sports and Exercise*, 47(4), 857–865. 10.1249/mss.0000000000000465 25137368

[ref28] Orchard, J., & Best, T. M. (2002). The management of muscle strain injuries: an early return versus the risk of recurrence. *Clinical Journal of Sport Medicine*, 12(1), 3–5. 10.1097/00042752-200201000-00004 11854581

[ref29] Šarabon, N., Marušič, J., Marković, G., & Kozinc, Ž. (2019). Kinematic and electromyographic analysis of variations in Nordic hamstring exercise. *PLoS One*, 14(10), e0223437. 10.1371/journal.pone.0223437 31644582 PMC6808554

[ref30] Schache, A. G., Wrigley, T. V., Baker, R., & Pandy, M. G. (2009). Biomechanical response to hamstring muscle strain injury. *Gait & Posture*, 29(2), 332–338. 10.1016/j.gaitpost.2008.10.054 19038549

[ref31] Sconce, E., Heller, B., Maden-Wilkinson, T., & Hamilton, N. (2021). Agreement between methods and terminology used to assess the kinematics of the Nordic hamstring exercise. *Journal of Sports Sciences*, 39(24), 2859–2868. 10.1080/02640414.2021.1968127 34459716

[ref32] Soga, T., Keerasomboon, T., Akiyama, K., & Hirose, N. (2022). Difference of Hamstring Activity Between Bilateral and Unilateral Nordic Hamstring Exercises With a Sloped Platform. *Journal of Sport Rehabilitation*, 31(3), 325–330. 10.1123/jsr.2021-0249 34969009

[ref33] Soga, T., Nishiumi, D., Furusho, A., Akiyama, K., & Hirose, N. (2021). Effect of Different Slopes of the Lower Leg during the Nordic Hamstring Exercise on Hamstring Electromyography Activity. *Journal of Sports Science & Medicine*, 20(2), 216–221. 10.52082/jssm.2021.216 33948099 PMC8057708

[ref34] Timmins, R. G., Bourne, M. N., Shield, A. J., Williams, M. D., Lorenzen, C., & Opar, D. A. (2016). Short biceps femoris fascicles and eccentric knee flexor weakness increase the risk of hamstring injury in elite football (soccer): a prospective cohort study. *British Journal of Sports Medicine*, 50(24), 1524–1535. 10.1136/bjsports-2015-095362 26675089

[ref35] van der Horst, N., Smits, D. W., Petersen, J., Goedhart, E. A., & Backx, F. J. (2015). The preventive effect of the nordic hamstring exercise on hamstring injuries in amateur soccer players: a randomized controlled trial. *American Journal of Sports Medicine*, 43(6), 1316–1323. 10.1177/0363546515574057 25794868

[ref36] van Dyk, N., Behan, F. P., & Whiteley, R. (2019). Including the Nordic hamstring exercise in injury prevention programmes halves the rate of hamstring injuries: a systematic review and meta-analysis of 8459 athletes. *British Journal of Sports Medicine*, 53(21), 1362–1370. 10.1136/bjsports-2018-100045 30808663

[ref37] Van Hooren, B., & Bosch, F. (2017). Is there really an eccentric action of the hamstrings during the swing phase of high-speed running? part I: A critical review of the literature. *Journal of Sports Sciences*, 35(23), 2313–2321. 10.1080/02640414.2016.1266018 27937671

[ref38] Vicente-Mampel, J., Bautista, I., Martin-Rivera, F., Maroto Izquierdo, S., Hooren, B., & Baraja-Vegas, L. (2022). Effects of ankle position during the Nordic Hamstring exercise on range of motion, heel contact force and hamstring muscle activation. *Sports Biomechanics*, 23(11), 2359–2371. 10.1080/14763141.2021.2025416 35045792

[ref39] Wu, G., Siegler, S., Allard, P., Kirtley, C., Leardini, A., Rosenbaum, D., Whittle, M., D'Lima, D. D., Cristofolini, L., Witte, H., Schmid, O., & Stokes, I. (2002). ISB recommendation on definitions of joint coordinate system of various joints for the reporting of human joint motion--part I: ankle, hip, and spine. International Society of Biomechanics. *Journal of Biomechanics*, 35(4), 543–548. 10.1016/s0021-9290(01)00222-6 11934426

[ref40] Yeung, S., Suen, A., & Yeung, E. (2009). A prospective cohort study of hamstring injuries in competitive sprinters: Preseason muscle imbalance as a possible risk factor. *British Journal of Sports Medicine*, 43(8), 589–594. 10.1136/bjsm.2008.056283 19174411

[ref41] Zabaloy, S., Healy, R., Pereira, L. A., Tondelli, E., Tomaghelli, L., Aparicio, J. … & Loturco, I. (2025). A Randomized Controlled Trial of Unresisted vs. Heavy Resisted Sprint Training Programs: Effects on Strength, Jump, Unresisted and Resisted Sprint Performance in Youth Rugby Union Players. *Journal of Human Kinetics*, 95, 199–214. 10.5114/jhk/200121 39944984 PMC11812154

